# Plasma Membrane Cholesterol as a Regulator of Human and Rodent P2X7 Receptor Activation and Sensitization*

**DOI:** 10.1074/jbc.M114.574699

**Published:** 2014-10-03

**Authors:** Lucy E. Robinson, Mitesh Shridar, Philip Smith, Ruth D. Murrell-Lagnado

**Affiliations:** From the Department of Pharmacology, University of Cambridge, Tennis Court Road, Cambridge CB2 1PD, United Kingdom

**Keywords:** Cholesterol Regulation, Inflammation, Ion Channel, Lipid Raft, Mutagenesis, Patch Clamp, Purinergic Receptor, P2X7

## Abstract

P2X7 receptors are nonselective cation channels gated by high extracellular ATP, but with sustained activation, receptor sensitization occurs, whereby the intrinsic pore dilates, making the cell permeable to large organic cations, which eventually leads to cell death. P2X7 receptors associate with cholesterol-rich lipid rafts, but it is unclear how this affects the properties of the receptor channel. Here we show that pore-forming properties of human and rodent P2X7 receptors are sensitive to perturbations of cholesterol levels. Acute depletion of cholesterol with 5 mm methyl-β-cyclodextrin (MCD) caused a substantial increase in the rate of agonist-evoked pore formation, as measured by the uptake of ethidium dye, whereas cholesterol loading inhibited this process. Patch clamp analysis of P2X7 receptor currents carried by Na^+^ and *N*-methyl-d-glucamine (NMDG^+^) showed enhanced activation and current facilitation following cholesterol depletion. This contrasts with the inhibitory effect of methyl-β-cyclodextrin reported for other P2X subtypes. Mutational analysis suggests the involvement of an N-terminal region and a proximal C-terminal region that comprises multiple cholesterol recognition amino acid consensus (CRAC) motifs, in the cholesterol sensitivity of channel gating. These results reveal cholesterol as a negative regulator of P2X7 receptor pore formation, protecting cells from P2X7-mediated cell death.

## Teaser

**Background:** P2X7 receptors are important in inflammation and immunity, but excessive activation results in cell death.

**Results:** Facilitation of P2X7 receptor currents and dilation of the pore are enhanced by cholesterol depletion and reduced by cholesterol loading.

**Conclusion:** Plasma membrane cholesterol destabilizes the open and dilated states of the receptor pore.

**Significance:** Targeting of P2X7 to cholesterol-rich lipid rafts may protect cells from death.

## Introduction

P2X7 receptors are ATP-gated cation channels, highly expressed in cells of hematopoietic lineage, which play an important role in inflammation and immunity (1–3). In macrophages, the P2X7 receptor can trigger inflammasome activation and IL-1β secretion, production of reactive oxygen species, and killing of intracellular microorganisms (4, 5). The receptor is also found in endothelial and epithelial cells, where it regulates ion transport, secretion, cell survival, and apoptosis (6–9). P2X7 is up-regulated in many other tissues under pathological and pro-inflammatory conditions and can trigger cell death (10). These diverse and potentially severe outcomes of P2X7 activation mean that regulation of the receptor is crucial.

P2X7 is the low affinity P2X receptor that responds to pathologically high levels of extracellular ATP, thus mediating the effects of ATP as a danger signal (10, 11). It can, however, be partially ligated and activated by much lower levels of ATP, thereby contributing to normal physiological responses. Its ability to respond to a wide range of ATP concentrations can be explained by ATP binding to the three sites on the trimeric receptor with negative cooperativity (12). Partial occupancy results in opening of an intrinsic pore nonselective for small mono- and divalent cations including Ca^2+^, whereas full occupancy at high ATP concentrations will, with some delay, trigger the dilation of the pore to accommodate much larger organic cations. Once in this sensitized or facilitated state, the process reverses slowly and the receptor pore dilates more rapidly upon subsequent application of agonist, eventually leading to cell death. Thus, the rate and extent of P2X7 sensitization determine the outcome of receptor activation. The mechanisms that regulate P2X7 receptor sensitization are not well characterized, although species differences have been described; the human P2X7 receptor sensitizes considerably more slowly in response to repeated or prolonged agonist application than the rat and mouse forms, and although Ca^2+^/calmodulin binds to the rat receptor to promote sensitization, it does not regulate the human receptor (13, 14).

Like many other ionotropic receptors, the ATP-gated P2X receptors associate with lipid rafts, as shown by their low buoyant density in a sucrose density gradient following cell fractionation (15–20). These membrane microdomains, rich in cholesterol and sphingolipids, attract some proteins but exclude others, thus providing a platform for the organization of distinctive signaling complexes (21, 22). The targeting of P2X7 to rafts has been demonstrated for endogenous receptors in salivary gland cells and airway epithelial cells, as well as for heterologously expressed receptors in HEK 293 cells (17, 18, 23–26). Palmitoylation of cysteines within the distinctively long cytoplasmic C terminus of P2X7 is suggested to promote raft association (18), although it is unclear which of the 17 conserved cysteines within this region have this covalent modification. In airway epithelial cells, P2X7 is reported to form a complex with caveolin-1, a key component of specialized invaginated lipid rafts called caveolae (23, 24). Perturbation of lipid rafts may disrupt the assembly of signaling complexes, but it can also directly affect the activity of proteins within these domains (27). Cholesterol has been shown to play a critical role in regulating the activity of many integral membrane proteins including ion channels (28). For the P2X7 receptor, perturbation of lipid rafts has been shown to affect downstream signaling pathways, in particular those associated with changes in lipid metabolism (16), but the direct effect on channel activation and sensitization is unknown.

The role of lipid rafts in the regulation of P2X7 receptor function is of interest for several reasons. First, some cells that express P2X7 receptors, such as macrophages and lymphocytes, have poorly defined or absent caveolae, whereas others, such as epithelia, are highly enriched in caveolae, so it seems likely that the lipid environment of P2X7 receptors will differ in a cell type-dependent manner (27). Second, activation of P2X7 receptors increases ceramide production (16, 29, 30), which has the potential to remodel rafts and might contribute to activity-dependent regulation of P2X7 receptor activation. Third, perturbation of lipid rafts and changes in plasma membrane cholesterol are associated with inflammation (31), so given the key role of P2X7 in mediating pro-inflammatory actions of extracellular ATP, it is important to understand how these changes may affect P2X7 function in the innate immune response and in chronic inflammatory diseases.

Changes in P2X receptor function after acute perturbation of lipid rafts has been shown for P2X1, P2X3, and P2X4 receptors; cholesterol depletion with methyl-β-cyclodextrin (MCD)^4^ inhibits channel activation (15, 19, 32, 33). Interestingly, the inhibition of P2X1 receptor currents was prevented by prior incubation with a cytoskeletal stabilizing agent, jasplakinolide, implicating cytoskeletal changes in this inhibition (34). P2X2 receptor currents are insensitive to MCD-mediated cholesterol depletion, and this differential sensitivity of P2X1 and P2X2 receptors was attributed to a region within the N terminus close to the first transmembrane domain (15). In this study, we compared the effects of cholesterol depletion and cholesterol loading on human and mouse P2X7 receptor whole-cell currents and large pore formation. Unlike other P2X receptor subtypes, acute depletion of cholesterol enhanced agonist-evoked currents and membrane permeabilization, whereas cholesterol loading suppressed P2X7 receptor function. N- and C-terminal regions proximal to the transmembrane domains are involved in the cholesterol dependence of channel gating. Thus, cholesterol acts as a negative regulator of P2X7 receptor channel activation and sensitization to prevent excessive activation.

## EXPERIMENTAL PROCEDURES

### 

#### 

##### Reagents and Drugs

All reagents were purchased from Sigma-Aldrich unless otherwise stated. Drugs were made up in DMEM (Life Technologies), and cells were pretreated at 37 °C, 5% CO_2_ in a humidified incubator. MCD and α-cyclodextrin (αCD) were used at 5 mm for 15 min, and water-soluble cholesterol was used at 100 μg/ml for 30 min. Pan-caspase inhibitor, zVAD-FMK, was purchased from BioVision Inc. (Milpitas, CA) and used at 5 μm for 1 h. Latrunculin-A was used at 5 μm for 30 min. Jasplakinolide was used at 30 nm for 1 h, and (−)-blebbistatin was used at 100 μm for 1 h; both were purchased from the Cayman Chemical Company (Ann Arbor, MI). For bromopalmitate (BrP), a stock solution of 100 mm was made up fresh in DMSO each time and diluted 1:1000 in DMEM plus serum to give a final concentration of 100 μm for 16 h. For carbenoxolone treatment, cells were not pretreated, but 30 μm was present in all solutions used in the experiment. Rabbit polyclonal P2X7 C terminus antibody was obtained from Alomone Labs (Jerusalem, Israel), and anti-mouse and anti-rabbit HRP-conjugated secondary antibodies were from Thermo Fisher Scientific and Bio-Rad, respectively.

##### Cell Culture

HEK 293 cells and TSA 201 cells were maintained in DMEM supplemented with 10% fetal bovine serum, 100 units/ml penicillin, and 50 units/ml streptomycin and transfected using Lipofectamine® 2000 (Life Technologies) and calcium phosphate, respectively, as described previously (35). Bone marrow-derived macrophages were isolated from the femora of 5–6-week-old mice and cultured as described previously (36).

##### Dye Uptake

Transfected TSA 201 cells on glass coverslips were bathed in reduced divalent normal extracellular solution (RD NES; 149 mm NaCl, 0.8 mm KCl, 0.6 mm CaCl_2_, 0.08 mm MgCl_2_, 10 mm d-glucose, 10 mm HEPES, pH 7.3) and imaged using the 40× oil immersion objective on a confocal microscope at room temperature. Ethidium bromide (20 μm in RD NES) and BzATP (300 μm in RD NES, but 30 μm for mouse P2X7k) were added at the specified time points, and change in fluorescence was recorded using LSM510 software (Zeiss, Germany). Ethidium bromide was excited at 543 nm, and emission light was collected at wavelengths ≥560 nm. Using ImageJ (Wayne Rasband, National Institutes of Health), the fluorescence intensity of each transfected cell (enhanced GFP was co-transfected as a marker) was recorded as a function of time and the average per coverslip was calculated, and the average uptake in five untransfected cells on the same coverslip (which was consistently low) was subtracted from the average of each coverslip to remove any drug effects that were not due to P2X7 activation. The rate of uptake was determined between 10 and 50 s after agonist application.

##### Cholesterol Assay

Cholesterol and protein levels were measured using the Amplex® Red cholesterol assay kit (Life Technologies) and Pierce BCA protein assay kit (Thermo Fisher Scientific), respectively.

##### Electrophysiology

Standard whole-cell recordings were performed at room temperature using an Axopatch 200B amplifier (Molecular Devices, Sunnyvale, CA). Patch pipettes (5–10 megaohms) were pulled from borosilicate glass (1B150F-4; World Precision Instruments, Sarasota, FL). Whole-cell currents were low pass-filtered at 1 kHz. Picospritzer II (Parker, Pine Brook, NJ) was used to apply 300 μm BzATP made up in extracellular solution with 0.05% (w/v) fast green to visually ensure drug delivery. Transfected HEK 293 cells were voltage-clamped at −60 mV. The pipette solution was 10 mm KCl, 70 mm K_2_SO_4_, 1 mm MgCl_2_, 10 mm HEPES, 75 mm sucrose, pH 7.3. Cells were maintained in NES (140 mm NaCl, 5 mm KCl, 2 mm CaCl_2_, 1 mm MgCl_2_, 10 mm d-glucose, 10 mm HEPES, pH 7.3), and 300 μm BzATP in NES was applied for a duration of 5 s followed by 1-min washout. For *N*-methyl-d-glucamine (NMDG^+^) experiments, the pipette solution was 145 mm NaCl, 10 mm EGTA, 10 mm HEPES, pH 7.35, and cells were maintained in NMDG buffer (155 mm NMDG, 2 mm CaCl_2_, 10 mm glucose, 10 mm HEPES, pH 7.35).

##### Biotinylation of Surface Receptors

Transfected TSA 201 cells were rinsed twice with ice-cold PBS and then incubated with 0.2 mg/ml EZ-Link sulfo-NHS-LC-biotin (Thermo Fisher Scientific), freshly prepared in NES, for 1 h at 4 °C. Nonreactive biotin was quenched, and unbound biotin was removed by washing three times with stock buffer (25 mm Tris-HCl, 150 mm NaCl, 10 mm EDTA, pH 7.5). Cells were lysed in solubilization buffer (stock buffer, 1% Triton X-100, 1 mm PMSF, protease inhibitor cocktail (Roche Applied Science)) on a rotating wheel for 1 h at 4 °C and then centrifuged at 18,000 × *g*. A 50-μl aliquot of the supernatant was diluted in Laemmli sample buffer and stored as the “total” protein sample, whereas the remaining supernatant was added to 30 μl of washed Pierce® Streptavidin UltraLink® resin (Thermo Fisher Scientific) and incubated on a rotating wheel at 4 °C for 2 h. Resin was centrifuged at 10,000 × *g* and washed in stock buffer with 1% Triton four times, and then proteins bound to the resin were eluted by the addition of 50 μl of Laemmli sample buffer and stored as the “biotinylated” protein sample. Samples were then analyzed by SDS-PAGE and immunoblotting. Two wells of a 6-well plate of confluent cells were solubilized in 350 μl of solubilization buffer unless otherwise stated.

##### DNA Constructs

Mouse P2X7 bearing an extracellular FLAG tag (DYKDDDDK) between His^85^ and Ser^86^ was generated by two-stage PCR using the following pairs of sense and antisense primers: first, 5′-AGCATAAAGCTTGTCGCCACCATGCCGGCTTGCTGCAGCTGG-3′ and 3′-TTTGTCGTCGTCGTCTTTATAGTCGTGTCCTAACTTCGTCACCCCACC-5′, and second, 5′-GACTATAAAGACGACGACGACAAAAGCATCTTTGACACTGCAGACTAC-3′ and 3′-GCTATAGCGGCCGCTCAGTAGGGATACTTGAAGCC-5′. This was subcloned into the Clontech GFP-N1 vector, using the NotI site to excise the coding sequence for GFP.

The human P2X7 Δ18 amino acid mutant was generated by two-stage PCR using the following pairs of sense and antisense primers: first, 5′-AGGATAAAGCTTGTCGCCACCATGCCGGCCTGCTGCAGCTGC-3′ and 5′-GTAGTATTCGTTGTTACTGGAGTAAGTGTC-3′, and second, 5′-TACTCCAGTAACAACGAATACTACTACAGG-3′ and 5′-GCATTACTCGAGTCAGTAAGGACTCTTGAAGCC-3′. This was subcloned into the Life Technologies pcDNA3.1 vector, using the HindIII and XhoI sites.

Single amino acid changes were made using the QuikChange II site-directed mutagenesis kit (Stratagene, La Jolla, CA). The sequences of all amplified regions were verified using automated DNA sequencing (Source BioScience, Nottingham, UK).

##### Immunostaining

Live labeling of P2X7-FLAG was carried out in HeLa cells, using a similar protocol to that described previously (37, 38). 48 h after transfecting HeLa cells with the P2X7-FLAG receptor and enhanced GFP (as a marker of transfection), control cells and those preincubated with BrP (16 h), MCD, or αCD (5 mm for 15 min) were incubated with anti-FLAG antibody for 1 h in NES at 12 °C, washed five times, and fixed with 4% paraformaldehyde. Cells were then incubated in blocking buffer for 1 h and then incubated with anti-mouse Cy3-conjugated secondary antibody for 2 h at room temperature. Fluorescence was imaged using a Zeiss Axiovert LSM510 confocal microscope with ×63 objective. Identical acquisition parameters were used for image capture of individual experiments. Images were imported into ImageJ, transfected cells were outlined, and total pixel values were obtained. Experiments were repeated three times, and at least 40 cells analyzed each time.

##### Data Analysis

Line and bar graphs were generated using Microsoft Excel. Error bars represent the S.E. The unpaired Student's *t* test was used to test for significance, unless otherwise stated. The dose-response curve was generated using a nonlinear regression curve fit equation in GraphPad Prism 6. Prism was also used to generate scatter graphs and to perform the two-way analysis of variance test.

## RESULTS

### 

#### 

##### Acute Manipulation of Plasma Membrane Cholesterol Regulates P2X7 Receptor Large Pore Formation

**FIGURE 1.**
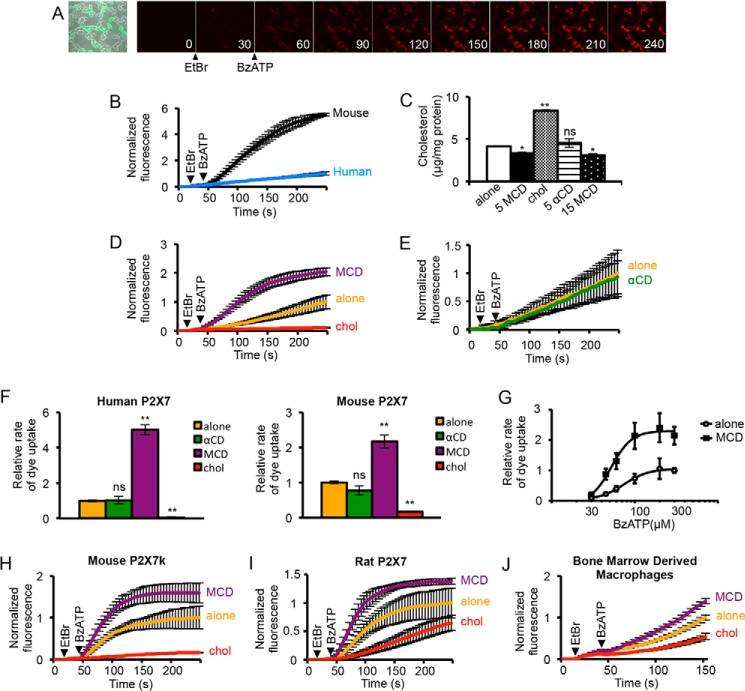
**P2X7 receptor-mediated dye uptake is enhanced by depletion of plasma membrane cholesterol and reduced by loading of cholesterol.** *A*, example confocal images captured during 245-s time series to measure dye uptake. Time of image capture, in seconds, is shown. Ethidium bromide was added at 20 s, and 300 μm BzATP was added at 45 s. The increase in fluorescence as a result of ethidium uptake (*red*) into P2X7-expressing cells (*green*) is apparent. *B*, human P2X7-mediated dye uptake was slower than mouse P2X7-mediated dye uptake (*n* = 3 coverslips, ∼10–30 cells per coverslip, *p* < 0.001), normalized to human P2X7. *C*, measurements of cholesterol (*chol*) in TSA 201 cells. A 15-min preincubation with 5 or 15 mm MCD reduced cholesterol, 5 mm αCD for 15 min had no effect, and 100 μg/ml water soluble cholesterol for 30 min increased cholesterol ∼2-fold (*n* = 3, *, *p* < 0.01, **, *p* < 0.001; *ns*, not significant). *D*, time course of human P2X7-mediated dye uptake, normalized to control. Uptake was enhanced by 5 mm MCD and inhibited by 100 μg/ml cholesterol (*n* = 3–4, *p* < 0.05). *E*, 5 mm αCD has no effect on human P2X7-mediated dye uptake, normalized to the untreated condition (*n* = 3, *p* > 0.05). *F*, rate of P2X7-mediated dye uptake 10–50 s after agonist application, normalized to control. Human P2X7 dye uptake was increased ∼5-fold by 5 mm MCD and almost abolished by 100 μg/ml cholesterol (*n* = 66 alone, *n* = 64 MCD and *n* = 6 cholesterol, **, *p* < 0.001). Mouse P2X7 dye uptake was increased ∼2.2-fold by MCD and reduced by cholesterol (*n* = 88 alone, *n* = 50 MCD and *n* = 4 cholesterol, **, *p* < 0.001). *G*, concentration-response curves for mouse P2X7 receptor-mediated dye uptake, with and without pretreatment with 5 mm MCD. Responses were normalized to the rate of increase in fluorescence in untreated cells stimulated with 300 μm BzATP. *H*, time course of dye uptake mediated by the more active mouse P2X7k variant, stimulated with 30 μm BzATP, normalized to the untreated condition (*n* = 3, *p* < 0.05 for MCD, *p* < 0.001 for cholesterol). *I*, rat P2X7 dye uptake was enhanced by 5 mm MCD, but not significantly (*p* = 0.07), and inhibited by 100 μg/ml cholesterol (*n* = 3, *p* < 0.05), normalized to the untreated condition. *J*, in mouse bone marrow-derived macrophages, dye uptake via endogenous P2X7 was potentiated by 5 mm MCD and reduced by 100 μg/ml cholesterol (*n* = 4, *p* < 0.001 for MCD, *p* < 0.01 for cholesterol), normalized to the untreated condition.

P2X7 receptor pore formation was assayed by the uptake of ethidium producing an increase in fluorescence as measured by confocal microscopy (Fig. 1*A*). For these experiments, mouse and human isoforms of P2X7 were expressed in TSA 201 cells, which are modified HEK 293 cells that express the receptors to a higher level. This improved the reliability of measuring dye uptake mediated by the human receptor. Receptors were activated by the application of the agonist BzATP in a reduced divalent extracellular solution. A concentration that produces a maximal rate of dye uptake at both receptors (300 μm) triggered a much slower rate of ethidium uptake for the human as compared with the mouse isoform consistent with a slower rate of pore dilation (Fig. 1*B*). To examine the effects of acute depletion of plasma membrane cholesterol, the cells were incubated with 5 or 15 mm MCD for 15 min prior to recording. An increase in cholesterol was achieved by 30 min of preincubation with 100 μg/ml cholesterol, made water-soluble by loading in MCD, and as control we used the analog αCD, which does not form a complex with cholesterol. Verification that these manipulations produced the expected changes in cholesterol content of cells was achieved using the Amplex red cholesterol assay (Fig. 1*C*). The decrease in cholesterol produced by a 15-min incubation with MCD was small but significant and might be limited by the relatively short duration of incubation as compared with other studies (15, 39). The αCD analog produced no change, as expected, whereas cholesterol-loaded MCD produced a significant increase in cholesterol. Following MCD treatment, the BzATP-evoked rate of rise in ethidium fluorescence increased by 5.0-fold for human P2X7 and 2.2-fold for mouse P2X7, whereas incubation with cholesterol-loaded MCD produced a profound inhibition and αCD had no effect (Fig. 1, *D–F*). Over a range of BzATP concentrations, MCD produced a large increase in the maximal rate of dye uptake with a small leftward shift in the EC_50_ value (52 μm with MCD *versus* 74 μm for control, for mouse P2X7) (Fig. 1*G*). Acute manipulations of cholesterol produced similar effects on the more active P2X7k mouse splice variant and on the rat P2X7 receptor (Fig. 1, *H* and *I*). Responses mediated by endogenous P2X7 receptors in mouse bone marrow-derived macrophages were also potentiated and inhibited by cholesterol depletion and loading, respectively (Fig. 1*J*). Thus, although other P2X receptors are inhibited by MCD treatment, the function of P2X7 receptors is enhanced.

**FIGURE 2.**
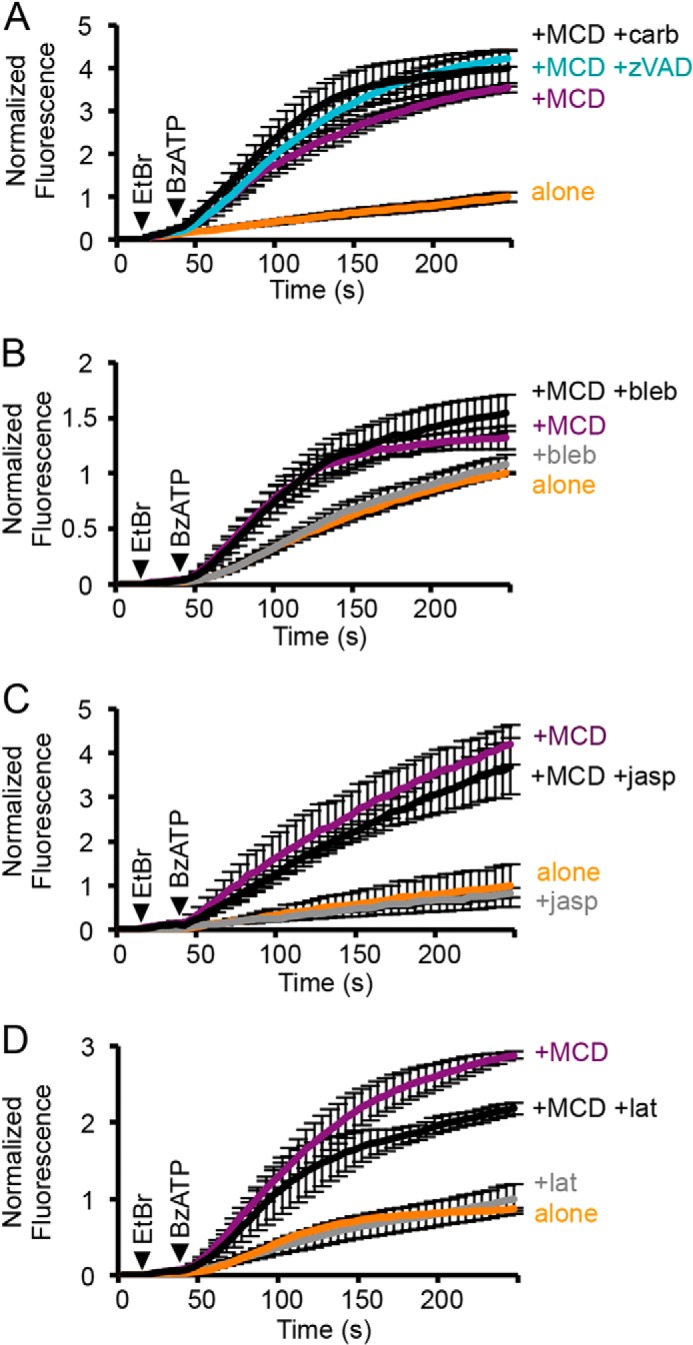
**The enhancement in P2X7 receptor-mediated dye uptake by MCD treatment is independent of pannexin-1 and cytoskeletal changes.** *A*, neither carbenoxolone (+*carb*; 30 μm) nor pretreatment with the caspase inhibitor zVAD-FMK (+*zVAD*; 5 μm for 1 h) reduced human P2X7-mediated ethidium uptake following MCD treatment. (*n* = 6, *p* > 0.05). *B*, preincubation with (−)-blebbistatin (+*bleb*; 100 μm for 1 h), an inhibitor of non-muscle myosin 2, had no effect on the rate of mouse P2X7-mediated dye uptake, with or without MCD pretreatment (*n* = 12, *p* > 0.05). *C*, the actin-stabilizing agent jasplakinolide (+*jasp*; 30 nm for 1 h) had no effect on human P2X7-mediated dye uptake with or without MCD treatment (*n* = 4–8, *p* > 0.05). *D*, latrunculin-A (+*lat*; 5 μm for 30 min), an inhibitor of F-actin polymerization, had no effect on the initial rate of mouse P2X7-mediated dye uptake, with or without MCD treatment (*n* = 3–6, *p* > 0.05).

Membrane permeabilization to cationic dyes, triggered by P2X7 receptor activation, has been reported to involve uptake via pannexin hemi-channels and to be regulated by non-muscle myosins (40, 41). To test whether pannexin-1 contributed to ethidium uptake following cholesterol depletion, cells were treated with a pannexin-1 inhibitor, carbenoxolone (30 μm), but this had no effect (Fig. 2*A*). We also tested the caspase inhibitor, zVAD-FMK, as caspase-dependent cleavage of the C terminus of pannexin-1 activates the pannexin pore (42), and saw no change in BzATP-evoked ethidium uptake after MCD treatment (Fig. 2*A*). The involvement of non-muscle myosin 2 was examined by treating cells with blebbistatin, a selective inhibitor of its ATPase activity, but BzATP-evoked ethidium uptake was unaltered both with and without MCD treatment (Fig. 2*B*). These results suggest that the P2X7 receptor itself is the likely target of cholesterol regulation. The inhibition of P2X1 receptor currents by MCD treatment is blocked by prior treatment with the actin-stabilizing agent, jasplakinolide. Neither jasplakinolide (30–50 nm) nor latrunculin-A, an inhibitor of F-actin polymerization, reduced the potentiating effects of MCD on P2X7 receptor-mediated dye uptake (Fig. 2, *C* and *D*). Thus, cytoskeletal changes are unlikely to be involved in the enhanced permeabilizing action of P2X7 following cholesterol depletion.

##### MCD-mediated Cholesterol Depletion Potentiates P2X7 Receptor Current Amplitude and Sensitization

**FIGURE 3.**
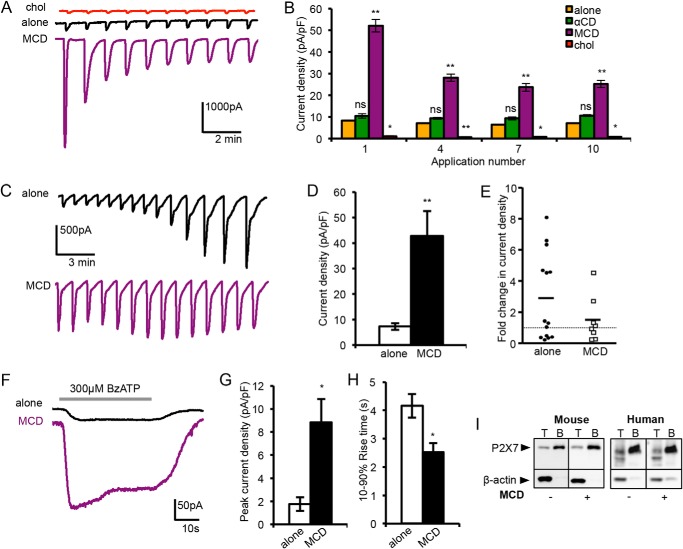
**Acute changes in cholesterol affect P2X7 whole-cell current amplitude, sensitization and NMDG permeability.** *A*, example traces of whole-cell patch clamp recordings from HEK 293 cells expressing the mouse P2X7 receptor and enhanced GFP, stimulated with repeated 5-s applications of 300 μm BzATP followed by a 1-min wash. *chol*, cholesterol. *B*, mean current densities after one or more applications of BzATP, in untreated cells or cells pretreated with 5 mm αCD, 5 mm MCD, or 100 μg/ml cholesterol (*n* = 3–18 cells, *, *p* < 0.01, **, *p* < 0.001; *ns*, not significant). *pF*, picofarads. *C*, example traces of whole-cell recordings from cells expressing the human P2X7 receptor, stimulated with repeated 5-s applications of 300 μm BzATP followed by a 1-min wash. *D*, mean current density upon first agonist application, with and without 5 mm MCD pretreatment (*n* = 18 cells, **, *p* < 0.001). *E*, -fold change in current density from first agonist application to last application (>10), where each point represents one cell. The *solid line* shows mean -fold change, and the *dotted line* is at 1. *F–H*, mouse P2X7 receptor currents in the NMDG^+^ extracellular solution, stimulated with 300 μm BzATP for 40 s. Mean peak current densities and the 10–90% rise time are shown (*n* = 7–9, *, *p* < 0.01). *I*, TSA 201 cells expressing mouse P2X7 or human P2X7 receptors were untreated or treated with 5 mm MCD for 15 min and then surface-labeled with sulfo-NHS-LC-biotin. For mouse P2X7, 3 wells of a 6-well plate of cells were then solubilized in 350 μl of solubilization buffer, whereas for human P2X7, a T75 flask of cells was solubilized in 1 ml. The Western blot shows the total lysate (*T*) and surface-biotinylated sample (*B*) blotted for P2X7 and β-actin. MCD treatment did not affect the proportion of P2X7 receptors at the surface.

Whole-cell currents mediated by the mouse and human P2X7 receptors differ in the degree and rate at which they sensitize in response to exposure to agonist, with human P2X7 showing a more pronounced and slower rate of sensitization (13). We tested the effects of depleting or loading cholesterol on currents activated by repetitive 5-s applications of 300 μm BzATP at both receptors. The mouse P2X7 receptor gave reproducible responses that were strongly inhibited by pretreatment with MCD loaded with cholesterol (Fig. 3, *A* and *B*). Treatment with MCD alone dramatically enhanced the amplitude of the current evoked by the first application of agonist and, although subsequent responses were also of larger amplitude, they showed some desensitization, unlike the untreated cells. For the human P2X7 receptor, preincubation with MCD also caused a large increase in the amplitude of the initial currents (Fig. 3, *C* and *D*). Under control conditions, recordings showed considerable variability in the degree of run-up (Fig. 3*E*). Approximately 40% showed a profound run-up after >10 applications of BzATP; for this group, the mean increase was ∼6.1-fold. Following MCD treatment, initial currents were already a similar magnitude to that seen after run-up of untreated cells, and only two recordings subsequently showed a modest run-up. Thus, for both mouse and human P2X7, the effect of MCD is to sensitize the receptor to the initial application of agonist.

To investigate whether depleting cholesterol promotes the receptor opening to a pore-dilated state, as suggested by the ethidium uptake experiments, NMDG^+^ was used as the extracellular charge-carrying ion. Mouse P2X7 receptor currents were activated by a 40-s BzATP application, with and without prior exposure to MCD. At a negative holding potential (−60 mV), we expected to see a small outward current followed by an inward current, as others have reported for the rat receptor, reflecting the delayed dilation of the pore to accommodate NMDG^+^ (12). Instead, for the control cells, there was either no current or a small and slowly activating inward current (Fig. 3*F*). Following MCD treatment, inward currents activated more rapidly and were of much larger amplitude, consistent with cholesterol depletion promoting a pore-dilated state of P2X7 (Fig. 3, *F–H*). To determine whether or not the increase in the peak current amplitude reflects a higher expression of the receptor at the plasma membrane, surface-exposed proteins were analyzed by biotinylation followed by Western blot. There was no change in either the biotinylated or the total levels of mouse and human P2X7 receptors following MCD treatment (Fig. 3*I*). Therefore the effects are on receptor activity.

##### An N-terminal Region Proximal to TMD1 Contributes to the Cholesterol Dependence of P2X7 Receptor-mediated Dye Uptake

**FIGURE 4.**
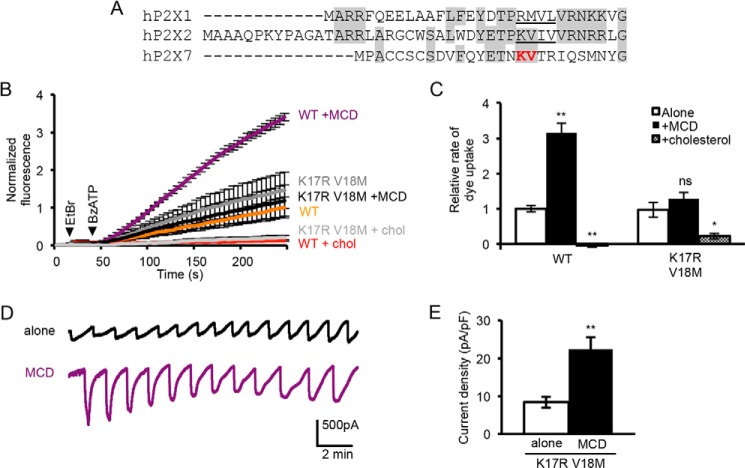
**The N terminus is important for the sensitivity of P2X7 receptor activity to cholesterol depletion.** *A*, amino acid sequence alignment of the N-terminal region of human P2X1, P2X2, and P2X7 receptors, with conserved amino acids highlighted in *gray*, P2X1 aa20–23 and equivalent residues in P2X2 *underlined*, and the P2X7 residues Lys^17^ and Val^18^ in *red. B*, time course of dye uptake mediated by the K17R/V18M mutant and wild type P2X7, alone, pretreated with MCD and loaded with cholesterol (*chol*), normalized to the untreated WT P2X7. *C*, bar graph of the relative rate of dye uptake normalized to the untreated WT P2X7. Dye uptake mediated by the K17R/V18M mutant is not potentiated by MCD, but is inhibited by cholesterol loading (WT: *n* = 14 alone, *n* = 11 MCD, *n* = 3 cholesterol; mutants: *n* = 12 alone, *n* = 11 MCD, *n* = 3 cholesterol; *, *p* < 0.05, **, *p* < 0.001; *ns*, not significant). *D*, whole-cell patch clamp recordings of human P2X7 K17R/V18M receptor ± 5 mm MCD pretreatment, stimulated with repeated 5-s applications of 300 μm BzATP followed by a 1-min wash. *E*, mean peak current density upon first application of BzATP. MCD pretreatment potentiates initial current density (*n* = 10–17 cells, **, *p* < 0.001), but the extent of potentiation is less than the wild type receptor (*p* < 0.05, two-way analysis of variance). *pF*, picofarads.

Regions of the P2X7 receptor involved in sensitivity to cholesterol were probed by mutagenesis analysis of the human variant as this showed the greatest potentiation of dye uptake by MCD. Previously, the differential sensitivity of P2X1 and P2X2 receptors to cholesterol depletion was attributed to a region within the middle of the N terminus, shown *underlined* in the alignment of the N-terminal regions (Fig. 4*A*) (15). Mutating residues 20–23 in P2X1 to those present in P2X2 (RMVL to KVIV) removed the inhibition of currents produced by MCD. The KV residues are common between P2X2 and P2X7, and so we tested the effect of the reverse mutations (K17R and V18M). This had no effect on the rate of ethidium uptake under control conditions but abolished the potentiation by MCD treatment (Fig. 4, *B* and *C*). Cholesterol-loaded MCD was, however, still effective in blocking dye uptake by this mutant, indicating that sensitivity to cholesterol was altered but not lost. This sensitivity was apparent for whole-cell currents, which showed a significant increase in amplitude following MCD treatment (Fig. 4*E*), although the ∼2-fold increase was less than half that seen with the wild type receptor. Currents were also inhibited by cholesterol loading (current density was 1.0 pA/pF and 2.5 pA/pF from two recordings). These results suggest that this N-terminal region contributes to the cholesterol sensitivity of the P2X7 receptor but that other residues or regions of the receptor are also involved in this process.

##### Cholesterol Sensing within the P2X7 Receptor C Terminus

**FIGURE 5.**
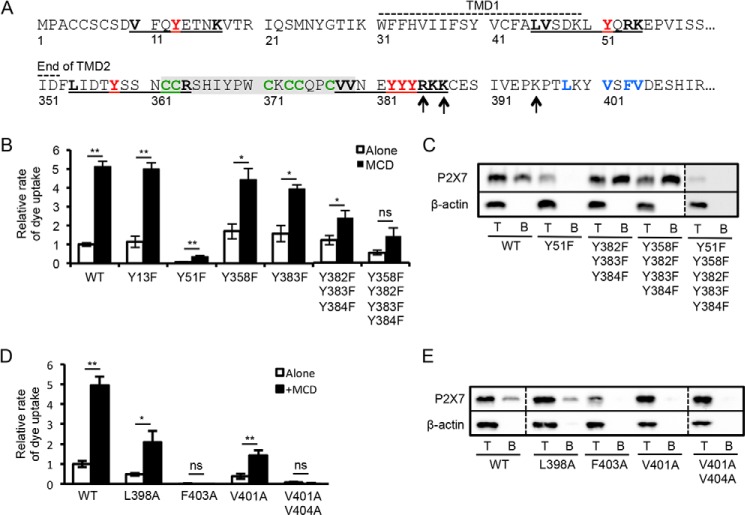
**Cholesterol-sensing within the proximal C terminus of the P2X7 receptor.** *A*, amino acid sequence of the human P2X7 N terminus and TMD1, and the end of the TMD2 and proximal C terminus. The CRAC motifs are *underlined*, with constituent residues in *bold* and central tyrosines in *red. Arrows* indicate the amino acids reported to interact with phosphatidylinositol 4,5-bisphosphate (48), and the cluster of hydrophobic residues downstream of these is in *bold/blue*. The 18-amino acid cysteine-rich region is highlighted in *gray*, and its cysteines are in *bold/green. B*, bar graph of the relative rates of dye uptake ± 5 mm MCD for P2X7 Tyr to Phe mutants normalized to the untreated WT P2X7 (WT: alone *n* = 16, MCD *n* = 5; mutants: *n* = 3–6; *, *p* < 0.01, **, *p* < 0.001; *ns*, not significant). *C*, cells expressing P2X7 WT or P2X7 mutants were surface-labeled with sulfo-NHS-LC-biotin. The Western blot shows the total lysate (*T*) and surface-biotinylated sample (*B*) blotted for P2X7 and β-actin. The Y51F mutation inhibited surface and total expression of the receptor. *D*, bar graph of the relative rates of dye uptake ± 5 mm MCD, for hydrophobic to alanine mutants normalized to the untreated WT P2X7 (WT: *n* = 9; mutants: *n* = 3–6; *, *p* < 0.05, **, *p* < 0.001). *E*, cells expressing P2X7 WT or P2X7 mutants were surface-labeled with sulfo-NHS-LC-biotin. The Western blot shows the total lysate (*T*) and surface-biotinylated sample (*B*) blotted for P2X7 and β-actin. Surface expression was inhibited by V401A, F403A, and V404A mutations.

The sensor for plasma membrane cholesterol will lie either within one of the transmembrane segments or in a region that dips into the membrane but does not necessarily traverse it. For example, the cholesterol-sensing region of large conductance calcium-activated potassium channel was recently shown to be in the cytosolic C terminus after TMD7 (43). The motif (L/V)*X*_1–5_Y*X*_1–5_(K/R) has been described as a cholesterol recognition amino acid consensus (CRAC) motif; it was first identified in a mitochondrial membrane transporter protein known as the peripheral-type benzodiazepine receptor and has since been identified in other proteins (44–46). Several tyrosine residues in the vicinity of the transmembrane domains of P2X7 lie within such a motif, including one within the N terminus adjacent to the KV residues (Tyr^13)^, a tyrosine at the extracellular end of TMD1 (Tyr^51^), a tyrosine at the cytoplasmic end of TMD2 (Tyr^358^), and then slightly farther downstream, a series of three adjacent tyrosine residues (Tyr^382^, Tyr^383^, and Tyr^384^) within overlapping motifs (Fig. 5*A*). The Y13F mutation had no effect on the rate of dye uptake, whereas the Y51F mutation abolished this response (Fig. 5*B*). Analysis of surface-biotinylated proteins showed no expression of this mutant at the plasma membrane and a reduction in total expression, consistent with its lack of function (Fig. 5*C*). This tyrosine is conserved in all P2X receptors, although only in P2X7 is the Tyr followed by positively charged residues at the +2 and +3 positions, hence conforming to the CRAC motif. The equivalent mutation in P2X4 (Y54F) is tolerated without loss of function, and even less conservative substitutions (*e.g.* Y54A) inhibit function but not plasma membrane expression (47). This suggests that in the P2X7 receptor, interactions involving this tyrosine are different and critical for the structure of the receptor.

Within the C terminus of P2X7, the individual Y358F and Y383F mutations had little effect on dye uptake, although the -fold increase produced by MCD treatment was reduced as compared with the wild type receptor (Fig. 5*B*). This -fold increase was further reduced in the triple mutant (Y382F/Y383F/Y384F), and when combined with Y358F, the potentiation by MCD did not reach significance (*p* > 0.05). There was no disruption in plasma membrane expression of these mutants; in fact surface expression appeared to be slightly increased (Fig. 5*C*). Within this background, the Y51F mutation again completely disrupted plasma membrane expression. These results suggest a concerted role for the multiple CRAC motifs within the proximal C terminus in contributing to the cholesterol sensitivity of the receptor. For this region to be directly sensing cholesterol, it would need to be intimately associated with the membrane. The neighboring positively charged residues (Arg^386^, Lys^388^, and Lys^395^) are reported to interact with phosphatidylinositol 4,5-bisphosphate (48), and downstream of this is a cluster of hydrophobic residues that could also be important for membrane interactions (Fig. 5*A*). The L398A mutation was tolerated without loss of receptor expression but reduced the rate of dye uptake (Fig. 5, *D* and *E*). Reducing hydrophobicity by substituting alanines at the Val^401^, Phe^403^, and Val^404^ positions did, however, inhibit the plasma membrane expression and function of the receptor (Fig. 5*D*,E), suggesting that hydrophobicity here is important for its correct folding and trafficking.

**FIGURE 6.**
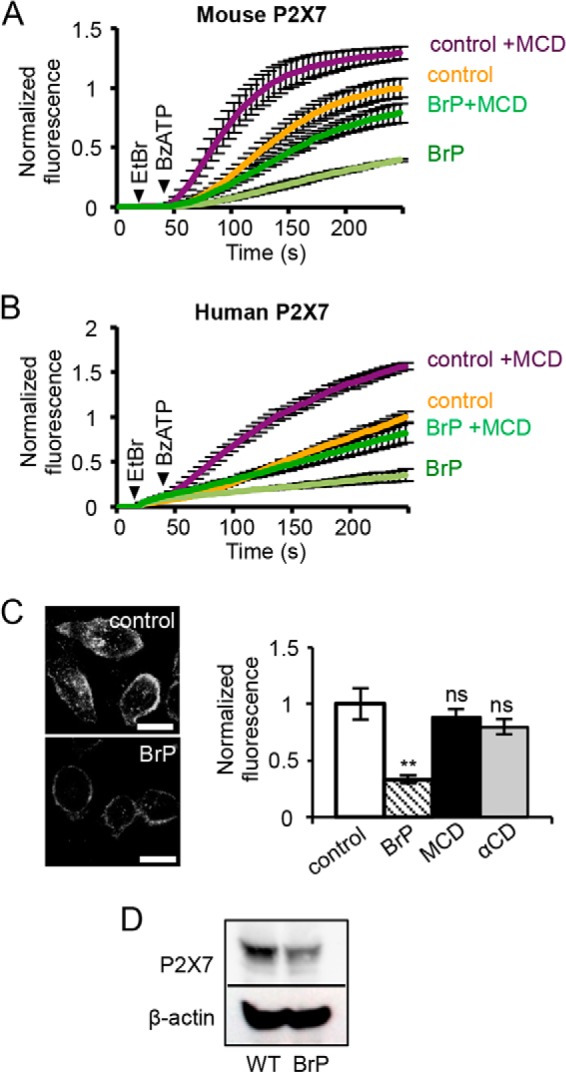
**Bromopalmitate pretreatment inhibits surface expression of the P2X7 receptor but does not abolish sensitivity to MCD.** *A* and *B*, time course of dye uptake by mouse P2X7 receptor (*A*) and human P2X7 receptor (*B*) after 16 h of pretreatment with 100 μm BrP or DMSO (control). P2X7 receptor-mediated dye uptake in BrP-treated cells was significantly potentiated by 5 mm MCD (*n* = 4 for mouse, *n* = 3 for human, *p* < 0.05). *C*, HeLa cells expressing mouse P2X7-FLAG receptors were immunostained by live labeling with anti-FLAG antibody. BrP reduced the surface expression of mouse P2X7-FLAG, but MCD treatment did not (*scale bar* = 20 μm, *n* = 32–40 cells per condition, **, *p* < 0.001; *ns*, not significant). *D*, Western blot of TSA 201 cells expressing mouse P2X7 shows that pretreatment with BrP reduces total expression of the receptor.

**FIGURE 7.**
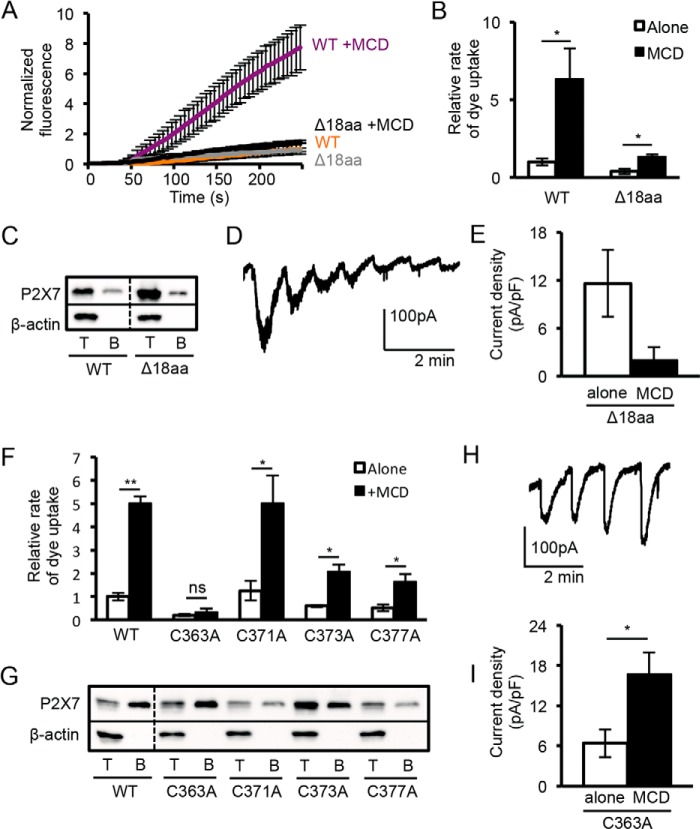
**The cysteine-rich region within the proximal C terminus of P2X7 is important for normal pore dilation.** *A*, time course of dye uptake mediated by P2X7 Δ18aa ± 5 mm MCD normalized to the untreated WT P2X7. *B*, relative rates of dye uptake mediated by P2X7 Δ18aa ± MCD, normalized to untreated WT P2X7 (WT: *n* = 6 alone, *n* = 2 MCD; mutants: *n* = 7 alone, *n* = 4 MCD; *, *p* < 0.05). *C*, Western blot shows that total (*T*) and surface-biotinylated (*B*) expression of P2X7 Δ18aa was similar to WT P2X7 (*panel B*). *D*, example whole-cell current recording for P2X7 Δ18aa stimulated with repeated 5 s applications of 300 μm BzATP. Currents had slow activation kinetics, exhibited run down, and were flickery. *E*, the mean peak current density for the first agonist application appeared to be reduced by MCD treatment, although this was not significant (alone *n* = 13, MCD *n* = 8, *p* > 0.05). *F*, bar graph of the relative rates of dye uptake ± 5 mm MCD for P2X7 Cys to Ala mutants, normalized to untreated WT P2X7. (WT: alone *n* = 8, MCD *n* = 11; Cys to Ala mutants: *n* = 3–6; *, *p* < 0.05, **, *p* < 0.001; *ns*, not significant). *G*, Western blot shows that surface expression of C363A and C373A mutants was similar to WT, whereas C371A and C377A mutants had reduced surface expression. *H*, whole-cell recording of P2X7 C363A receptor currents, stimulated with repeated 5-s applications of 300 μm BzATP. *I*, P2X7 C363A mean peak current density upon first application of BzATP was potentiated by 5 mm MCD pretreatment (*n* = 5, *, *p* < 0.05).

Immediately upstream of the Tyr^382–384^ CRAC motifs is a series of cysteine residues that are potential sites of palmitoylation (Fig. 5*A*). A reduction in palmitoylation and targeting to rafts are expected to reduce the sensitivity of the receptor to MCD treatment. To investigate the role of palmitoylation in cholesterol sensitivity, we initially tested the effects of BrP, an inhibitor of palmitate incorporation, on receptor function and cholesterol sensitivity. Following a 16-h preincubation with BrP, the rate of dye uptake decreased although it was still potentiated by MCD treatment (Fig. 6, *A* and *B*). There was also an ∼60% drop in plasma membrane expression of the receptor and a reduction in its overall expression, which would account for the reduced dye uptake response (Fig. 6, *C* and *D*). These effects of BrP are not readily interpretable and could also be off-target. As an alternative approach, the effect of removing these potential palmitoylation sites was tested by deleting the juxtamembrane cysteine-rich region (Δ18aa). In the rat P2X7 receptor, deletion of this region accelerates receptor sensitization but has no effect on dye uptake (12, 49). Surprisingly, the equivalent deletion in human P2X7 (Δ362–379) inhibited dye uptake and its potentiation by MCD (Fig. 7, *A* and *B*). Surface biotinylation indicated that the Δ18aa mutant was still expressed at the plasma membrane at a similar level as the wild type receptor (Fig. 7*C*). Currents mediated by P2X7 Δ18aa were small with unusually slow kinetics of activation and “flickery“ in nature, suggesting that the pore was rapidly oscillating between an open and shut state (Fig. 7*D*). The currents ran down rather than sensitized with repeated application of agonist, and MCD treatment reduced rather than enhanced the amplitude of the initial response to BzATP application (Fig. 7*E*). These results indicate the importance of this juxtamembrane region for normal pore gating and in conferring cholesterol sensitivity to this process. Individual cysteine to alanine mutations within this region were also made in an attempt to identify a critical cysteine residue. The C371A mutation had no effect on ethidium uptake, C373A and C377A mutations inhibited uptake without abolishing sensitivity to MCD treatment, and the C363A mutation abolished uptake with or without MCD treatment (Fig. 7*F*). All mutants were expressed at the cell surface, and the C363A mutant carried an inward current that showed some sensitization and was potentiated by MCD (Fig. 7, *G–I*). Our conclusion is that the C363A mutation disrupts pore dilation and dye uptake via a mechanism independent of cholesterol sensitivity. We failed to identify a key role for any one cysteine in conferring sensitivity to acute cholesterol depletion, but this is consistent with the findings of Gonnord *et al.* (18), which suggest that multiple cysteine residues within the P2X7 C terminus are palmitoylated.

## DISCUSSION

The P2X7 receptor is unusual for an ion channel in that it has two phases of activation; it opens initially to a cation-conducting pore that allows small mono- and divalent cations to permeate, but in response to prolonged or repetitive application of agonist, it undergoes a sensitization process, resulting in increased current amplitudes and dilation of the pore with potentially harmful consequences for the fate of the cell (12, 50). In this study, we identify plasma membrane cholesterol as a key regulator of human and rodent P2X7 receptor sensitization and pore dilation. The effects of cholesterol depletion and cholesterol loading on P2X7 receptor function indicate that cholesterol acts to suppress opening of the channel pore, particularly its large pore, and thus limits excessive activation. This appears to be particularly relevant for the human P2X7 receptor, which normally mediates a much slower rate of dye uptake than the rat or mouse isoforms. Therefore P2X7 receptors localized to cholesterol-rich lipid rafts are expected to exhibit a more reluctant mode of gating, and so targeting of receptors to rafts may be a means of limiting the extent of receptor activation as well as organizing downstream signaling. Disruption of lipid rafts, which we achieved with MCD treatment, has been shown to occur in chronic inflammation (31), and so P2X7 receptor function may be enhanced in these conditions, with associated pore dilation and cell death potentially contributing to the pathogenesis of chronic inflammatory diseases.

Our finding that MCD treatment potentiates P2X7 receptor function contrasts with its inhibitory effects on other P2X receptor subtypes including P2X1, P2X3, and P2X4 (15, 19, 32). It is also not entirely consistent with a previous study on rat submandibular gland cells, in which preincubation with MCD reduced ATP-stimulated phospholipase A_2_ activity, but did not affect the rise in cytosolic calcium (16). The study concluded that opening of the P2X7 channel pore is unaffected by disrupting rafts, but coupling between the P2X7 receptor and sphingomyelinase and ceramide generation occur preferentially within rafts. Our study provides a much more direct measure of the activation and dilation of the P2X7 receptor pore. It is likely that calcium responses evoked by ATP in submandibular gland cells are not solely mediated by the P2X7 receptor, which is why they were unaffected by MCD treatment. Sphingomyelin and sphingomyelinase are concentrated within rafts, and therefore generation of ceramide is expected to occur preferentially within these microdomains (22, 27). Thus, perturbation of lipid rafts could concomitantly enhance the efficacy of agonist-stimulated pore opening and dilation while disrupting the efficiency of coupling between the receptor and lipid metabolism pathways.

Mutational analysis of the P2X7 receptor supports a role for both N-terminal and C-terminal regions proximal to the transmembrane domains in the cholesterol sensitivity of channel gating. The region within the N terminus is equivalent to that shown to regulate the sensitivity of the P2X1 receptor to MCD treatment (15). Previous studies also highlight this region as playing an important role in the activation and sensitization of the P2X7 receptor. For example, threonine at position 15, just upstream of the mutated lysine 17 and valine 18, when substituted for either glutamate or lysine, promotes the pore-dilated state of rat P2X7 as measured by NMDG permeability (12). Within the proximal C terminus of P2X7 are four CRAC motifs, and multiple Tyr to Phe mutations within these motifs progressively lead to a reduction in MCD sensitivity. In a recent study, multiple CRAC motifs within the C terminus of the BK-type potassium channel were shown to contribute to the cholesterol sensitivity of channel activity (43). Although CRAC motifs are common, and the presence of a motif does not necessarily imply that a protein binds cholesterol (51), close proximity of the motif to the plasma membrane may be an important determinant. For example, the CRAC motif reported to regulate cannabinoid type 1 receptor cholesterol sensitivity is at the end of TMD7, and in the HIV-1 gp41 protein, the CRAC motif, which binds cholesterol, is found immediately adjacent to the membrane-spanning domain (45, 46). There is also evidence that sterols are mainly in the cytoplasmic leaflet of the bilayer, suggesting that regions of proteins located at the membrane-cytosol interface are well placed for sensing cholesterol (52). We propose that the proximal C-terminal region of P2X7 is in contact with and may dip back into the plasma membrane, via palmitoyl groups and hydrophobic residues. Comparing this region with the proximal C terminus of the other P2X receptor subtypes, the 18-amino acid cysteine-rich region is unique to P2X7, but there are similarities in the sequences downstream of this. Similar to P2X7, P2X4 has three adjacent tyrosines, whereas P2X1 has two. These are located immediately downstream of TMD2, and in both receptors are preceded and followed by clusters of basic residues that are involved in binding membrane phosphoinositides (53). In P2X1, these two tyrosines lie within a sequence that conforms to two overlapping CRAC motifs (Leu^357^-*X*_4_-Tyr^362^-Tyr^363^-*X*_2_-Lys^366^-Lys^367^). Whether or not this region is involved in the cholesterol sensitivity of P2X1 remains to be established.

Although we favor the idea that cholesterol binds to P2X7 to regulate its activity, an alternative possibility is that manipulating cholesterol levels changes the ordered structure and arrangement of lipids around the receptor, thereby altering its gating properties. For example, a reduction in cholesterol is expected to increase membrane fluidity, which could act to promote pore dilation, whereas increasing cholesterol and the rigidity of the membrane would have the opposite effect (22). The mutational analysis would then indicate that proximal N- and C-terminal regions are important in coupling changes in fluidity to the gating and dilation of the pore. The idea that the gating of the P2X7 receptor channel is particularly sensitive to the ordered nature of its lipid environment is supported by previous studies that looked at the effect of lysolipid products of phospholipase A_2_ on P2X7 receptor function and showed an enhancement of function, whereas other P2X receptor subtypes were relatively insensitive (54). Several lipids were examined, and those that enhanced receptor function were amphiphilic and capable of integrating into lipid bilayers, thereby disrupting its ordered structure and increasing fluidity. The crystal structure of the P2X4 receptor in its ATP-bound open state shows relatively few inter-subunit interactions in the transmembrane domains, which is consistent with the idea that the stability of this state, and even more so the dilated pore state of P2X7, will be sensitive to the nature of the surrounding lipids (55) (Protein Data Bank (PDB) number 4DW1).

In general, plasma membrane cholesterol is tightly regulated on a cellular level, through control of biosynthesis, efflux, and influx. However, localized, acute changes in cholesterol are likely to occur upon activation of signaling pathways linked to lipid metabolism. Ceramide levels have been shown to increase downstream of P2X7 receptor activation, via the action of sphingomyelinases, and by *de novo* ceramide generation (16, 29, 30). Ceramide displaces cholesterol from rafts and increases membrane fluidity (27, 56), which might contribute to stabilization of the dilated state of the P2X7 receptor pore. This leads to the hypothesis that perturbations in bilayer structure in the immediate environment of the receptor contribute to its activity-dependent sensitization. In addition to acute changes in the lipid environment of the receptor, longer-term changes might occur as a consequence of dysregulation of the normal cholesterol homeostatic mechanisms of the cell. For example, plasma membrane cholesterol levels increase with aging, which correlates with impairment in the innate immune response (57). In intestinal epithelial cells, cholesterol accumulates in response to exposure to *Salmonella*, leading to activation of anti-inflammatory pathways and protection of these cells from apoptosis (58). Also in intestinal epithelial cells, lipid raft disruption was shown to occur early in the inflammatory cascade in both mouse and human colitis, leading to intestinal epithelial barrier disruption (31). Given the role of P2X7 receptor in inflammation and apoptosis, it is tempting to speculate that dysregulation of the P2X7 receptor as a result of disruption of cholesterol homeostasis may play a role in some of these scenarios.

In conclusion, we have shown that changes in plasma membrane cholesterol affect the activation and sensitization of the P2X7 receptor; the rate of pore dilation is enhanced by depletion of cholesterol and strongly suppressed by an increase in cholesterol. This mode of regulation is particularly evident for the human form of the receptor, which sensitizes more slowly than the rodent isoforms. The targeting of P2X7 receptors to cholesterol-rich lipid rafts is likely to be an important mode of receptor regulation, whereas disruption of cholesterol homeostasis may promote P2X7 receptor-mediated membrane permeabilization and subsequent cell death in disease states.

## Footnotes

4 The abbreviations used are:

MCD: methyl-β-cyclodextrin
αCD: α-cyclodextrin
BrP: bromopalmitate
NES: normal extracellular solution
RD NES: reduced divalent normal extracellular solution
NMDG^+^: *N*-methyl-d-glucamine
TMD: transmembrane domain
CRAC: cholesterol recognition amino acid consensus
BzATP: 3′-*O*-(4-benzoyl)-benzoyl adenosine 5′-triphosphate
DMSO: dimethyl sulfoxide
aa: amino acids
z: benzyloxycarbonyl
fmk: fluoromethylketone
pF: picofarads.
